# High Electron Mobility in Si-Doped Two-Dimensional β-Ga_2_O_3_ Tuned Using Biaxial Strain

**DOI:** 10.3390/ma17164008

**Published:** 2024-08-12

**Authors:** Hui Zeng, Chao Ma, Meng Wu

**Affiliations:** 1 College of Science, Hunan University of Science and Engineering, Yongzhou 425199, China; 2 College of Materials Science and Engineering, Hunan University, Changsha 410082, China; 3 Fujian Provincial Key Laboratory of Semiconductors and Applications, Collaborative Innovation Center for Optoelectronic Semiconductors and Efficient Devices, Department of Physics, Xiamen University, Xiamen 361005, China

**Keywords:** first-principles calculations, 2D β-Ga_2_O_3_, biaxial strain, electronic structure, transport properties

## Abstract

Two-dimensional (2D) semiconductors have attracted much attention regarding their use in flexible electronic and optoelectronic devices, but the inherent poor electron mobility in conventional 2D materials severely restricts their applications. Using first-principles calculations in conjunction with Boltzmann transport theory, we systematically investigated the Si-doped 2D β-Ga_2_O_3_ structure mediated by biaxial strain, where the structural stabilities were determined by formation energy, phonon spectrum, and ab initio molecular dynamic simulation. Initially, the band gap values of Si-doped 2D β-Ga_2_O_3_ increased slightly, followed by a rapid decrease from 2.46 eV to 1.38 eV accompanied by strain modulations from −8% compressive to +8% tensile, which can be ascribed to the bigger energy elevation of the σ* anti-bonding in the conduction band minimum than that of the π bonding in the valence band maximum. Additionally, band structure calculations resolved a direct-to-indirect transition under the tensile strains. Furthermore, a significantly high electron mobility up to 4911.18 cm^2^ V^−1^ s^−1^ was discovered in Si-doped 2D β-Ga_2_O_3_ as the biaxial tensile strain approached 8%, which originated mainly from the decreased quantum confinement effect on the surface. The electrical conductivity was elevated with the increase in tensile strain and the enhancement of temperature from 300 K to 800 K. Our studies demonstrate the tunable electron mobilities and band structures of Si-doped 2D β-Ga_2_O_3_ using biaxial strain and shed light on its great potential in nanoscale electronics.

## 1. Introduction

Bulk β-gallium oxide (β-Ga_2_O_3_) possesses an ultrawide band gap (4.8 eV); however, its low mobility significantly limits its applications [1,2,3]. Doping engineering is one effective method that is used to tune the conductivity of wide-band gap semiconductors such as β-Ga_2_O_3_ [4,5,6,7,8]. Among plentiful dopants, the Si dopant is peculiar since it is not only one of the unintentionally introduced impurities during synthesis and device fabrication but also plays a vital role in modifying electron mobility, as reported in three-dimensional (3D) β-Ga_2_O_3_ [9]. For Si-doped 3D β-Ga_2_O_3_, electron mobilities of 0.1–196 cm^2^ V^−1^ s^−1^ have been reported. Baldini et al. studied the electron mobility of Si-doped homoepitaxial β-Ga_2_O_3_ films ranging from ~50 cm^2^ V^−1^ s^−1^ for a doping concentration of *n* = 8 × 10^19^ cm^−3^ to ~130 cm^2^ V^−1^ s^−1^ for a doping concentration of *n* = 1 × 10^17^ cm^−3^ [10]. Hernandez et al. illustrated that Si-doped homoepitaxial β-Ga_2_O_3_ films grown by chemical vapor deposition were characterized by electron mobilities in the range of 0.59–30.59 cm^2^ V^−1^ s^−1^ for Si concentrations ranging from 89.2 to 17.8 nmol/min [11]. Recently, Bhattacharyya et al. reported an electron mobility of 196 cm^2^ V^−1^ s^−1^ (*n* = 2.3 × 10^16^ cm^−3^) measured in a Si-doped β-Ga_2_O_3_ film grown on Fe-doped β-Ga_2_O_3_ substrates at 810 °C, which is the highest electron mobility value ever reported in β-Ga_2_O_3_ epitaxial films, suggesting the great potential of Si dopants in tuning the electron mobility of 3D β-Ga_2_O_3_ [12]. For these reported heteroepitaxial films, Zhang et al. indicated that the electron mobility was 0.1, 0.1, 0.5, and 5.5 cm^2^ V^−1^ s^−1^ for 0, 1.1, 4.1, and 10.4% Si-doped β-Ga_2_O_3_ thin films on sapphire substrates prepared using pulsed laser deposition (PLD) at 500 °C, respectively [13]. Khartsev et al. prepared high-quality Si-doped β-Ga_2_O_3_ films on sapphire substrates using PLD and reported an electron mobility of about 2.9 cm^2^ V^−1^ s^−1^ for *n* = 2.5 × 10^19^ cm^−3^ [14]. Wong et al. reported electron mobilities of 90–100 cm^2^ V^−1^ s^−1^ at carrier concentrations in the low to mid 10^17^ cm^−3^ range in β-Ga_2_O_3_ (010) using Si-ion implantation doping and molecular beam epitaxy [15]. 

Two-dimensional (2D) semiconductors have attracted much attention and are widely used in electronic and optoelectronic devices [16,17,18,19,20,21,22]. In the past few years, research interest has been mostly focused on graphene [23], transition metal dichalcogenides (TMDs) [24], and other materials in order to design new nanoelectronic devices, including solar cells and field effect transistors. Although the 2D materials mentioned above usually possess outstanding properties, some of their disadvantages, such as the zero band gap of graphene and the low carrier mobilities of TMDs, make it temporarily difficult to adopt multifunctional applications. Therefore, the discovery and engineering of novel 2D materials with moderate band gaps and high carrier mobility are worthy of research focus. Lately, the fabrication of 2D β-Ga_2_O_3_ was reported using mechanical exfoliation, molecular beam epitaxy, and chemical synthesis methods, and its benefits stemmed from its fragile Ga-O bonds along the β-Ga_2_O_3_ [100] direction [25,26]. Owing to its lower-dimensional nature and its high surface-to-volume ratio, 2D β-Ga_2_O_3_ exhibits better physical properties and enormous potential for novel applications. For instance, a monolayer of β-Ga_2_O_3_ passivated by H possessed electron mobilities as high as 2685 cm^2^ V^−1^ s^−1^ and 156 cm^2^ V^−1^ s^−1^ along the b and c directions, respectively, based on theoretical studies [27]. The features of 2D β-Ga_2_O_3_-based solar-blind photodetectors were boosted to nearly three times higher than that of bulk β-Ga_2_O_3_ [28,29,30]. Therefore, 2D β-Ga_2_O_3_ has great potential for innovative nanoscale applications because it combines the benefits of outstanding electron mobility with affordable production techniques.

However, both theoretical and experimental efforts devoted to modifying the carrier mobility in Si-doped 2D β-Ga_2_O_3_ materials are lacking. Only limited reports showed the preferred Si atom occupation of the tetrahedral Ga site, which resulted in donor doping behaviors in Si-doped 2D β-Ga_2_O_3_ [31]. Especially considering the different local crystal structures of 2D and 3D β-Ga_2_O_3_, the results presented regarding 3D β-Ga_2_O_3_ are difficult to directly apply to 2D systems, which motivated us to comprehensively analyze the effects of Si dopants on the electronic and transport properties of 2D β-Ga_2_O_3_ here. Additionally, previous work showed that the 2D β-Ga_2_O_3_ is characterized by low elastic constants; therefore, systematic strain-modulated band structure investigations of 2D β-Ga_2_O_3_ shed light on its potential for use in the fabrication of flexible electronic devices [32]. Inspired by these concepts, we carried out first-principles calculations using the Perdew–Burke–Ernzerhof (PBE) functional and the Boltzmann transport theory to explore the strain-modulated transport properties of Si-doped 2D β-Ga_2_O_3_. Our work demonstrates that the electron mobilities and band structures of Si-doped 2D β-Ga_2_O_3_ are tunable using biaxial strain, highlighting its great potential for use in nanoscale electronics. 

## 2. Calculation Methods

### 2.1. Computational Details

**Figure 1:**
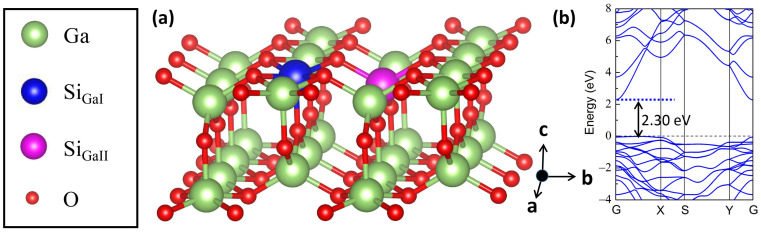
(**a**) The model of Si-doped 2D β-Ga_2_O_3_ structure. The O and Ga atoms are represented by the red and green spheres, respectively. The replaced GaI and GaII doping positions with a Si atom are indicated by the blue and magenta colors, respectively. (**b**) Band structure of undoped 2D β-Ga_2_O_3_ unit cell calculated by PBE functional. In this work, G (0, 0, 0), X (0.5, 0, 0), S (0.5, 0.5, 0), and Y (0, 0.5, 0) are set as the high symmetry points, respectively.

Vienna ab initio Simulation Package (VASP) Standard Edition 6.4 was used for first-principles calculations [33,34]. The generalized gradient approximation (GGA) of the PBE functional was adopted [35,36]. In this study, a 3 × 2 × 1 2D β-Ga_2_O_3_ supercell including 60 atoms was modeled. The criteria of energy cutoff, energy convergence, and residual forces were 450 eV, 1 × 10^−6^ eV/atom, and 0.01 eV/Å, respectively. The k-point grid was set at a resolution of 0.02 × 2π Å^−1^. The vacuum thickness was set at 15 Å along the *c*-axis direction to prevent the spurious interactions between layers. Bulk β-Ga_2_O_3_ contains two different Ga coordinations, i.e., octahedral GaI and tetrahedral GaII. Along the β-Ga_2_O_3_ [100] direction, bulk β-Ga_2_O_3_ can be cleaved into 2D β-Ga_2_O_3_ with a low cleavage energy [37]. After cleaving from 3D β-Ga_2_O_3_, as shown in Figure 1a, the GaII maintained four coordinations, whereas the GaI changed from six coordinations in 3D β-Ga_2_O_3_ to five coordinations in 2D β-Ga_2_O_3_. Thus, one 2D β-Ga_2_O_3_ is endowed with two inequivalent Ga sites, namely the square pyramidal GaI and the tetrahedral GaII. The ionic radius of Si^4+^ (0.040 nm) dopant is slightly lower than that of host Ga^3+^ (0.062 nm), which makes it difficult to be in the interstitial position and easier to substitute Ga atoms. Therefore researchers have focused on its substitution style instead of interstitial doping in 3D and 2D β-Ga_2_O_3_ systems theoretically [31,38,39] and experimentally [12] in the literature. In this study, one Si impurity was substituted at the GaI and GaII cation sites labeled as Si_GaI_ and Si_GaII_, respectively, as illustrated in Figure 1a. Furthermore, in this work, the *x* and *y* directions correspond to the crystalline a and b directions, respectively.

The dynamical stability was evaluated by phonon dispersion based on density functional perturbation theory (DFPT) within a 6 × 2 × 1 supercell [40]. The structural convergence criteria of energy and force were 10^−8^ eV and 10^−7^ eV/Å, respectively. To gain thermodynamical stability, ab initio molecular dynamic (AIMD) simulation was adopted within a 6 × 2 × 1 supercell. The electron mobility and relaxation time were determined by the deformation potential (DP) theory [41]. The Boltzmann theory with the BoltzTraP2 code was utilized to determine the electrical conductivity [42]. 

### 2.2. Formation Energies and Carrier Mobility Calculations

The formation energy $E_{f}$ of Si-doped 2D β-Ga_2_O_3_ is defined as [43,44]

(1) $$E_{f}=E_{defect}-E_{Ga_{2}O_{3}}-\sum_{i}n_{i}\mu_{i}$$

where $E_{defect}$ and $E_{Ga_{2}O_{3}}$, respectively, represent the energy of the Si-doped and pure 2D β-Ga_2_O_3_ configuration. $n_{i}$ demonstrates the quantity of i atom added ($n_{i}<0$) or extracted ($n_{i}>0$) from the pure β-Ga_2_O_3_ system. The chemical potential of $\mu_{\mathrm{Si}}$ and $\mu_{O}$ is gained from the stable bulk Si and O_2_, respectively. The related chemical potential should meet the boundary conditions and fall into the O-rich and Ga-rich cases in terms of the growth conditions, as stated in our previous works [5,43]. 

Based on the DP theory, the carrier mobility μ is calculated as follows [45]:

(2) $$\mu =\frac{2eh^{3}C_{2D}}{{\left(2\pi \right)}^{3}\times 3k_{B}T{\left|m*\right|}^{2}E_{1}^{2}}$$

where k_B_, h, e, and T are the Boltzmann constant, Planck constant, electric charge, and the temperature (300 K), respectively. The electron mobility μ and relaxation time τ are determined by the deformation potential (DP) theory, where three inconstant parameters are necessary, i.e., the deformation potential constant E_1_, elastic stiffness C_2D_, and the electron effective mass m_e_*. Deformation potential constant E_1_ is calculated by $E_{1}=\partial E_{edge}/\partial (\Delta a/a_{0})$, which denotes the shift of the band edge accompanied by uniaxial strain. Here, $E_{edge}$ illustrates the shifted energy of the conduction band minimum (CBM), $\Delta a/a_{0}$ is the variation in the lattice constant along a certain strained direction, and elastic stiffness C_2D_ is calculated by $C_{2D}=[\partial^{2}E/\partial {\left(\Delta a/a_{0}\right)}^{2}]/S_{0}$. Here, E is the total energy of the strained optimized supercell, and $S_{0}$ is the surface area of the strained structure. The electron effective mass m_e_* is calculated based on the equation as $m_{e}^{*}=\hbar^{2}/(\partial^{2}E/\partial k^{2})$ by fitting the band curves close to the CBM, and k is the electron wave vector. The CBM and valence band maximum (VBM) values are obtained from the band structures of Si-doped 2D β-Ga_2_O_3_. The relaxation time is estimated by $\tau =\mu m_{e}^{*}/e$.

## 3. Results and Discussion

### 3.1. Structural Stability

Pure 2D β-Ga_2_O_3_ possesses the lattice constants of a = 2.97 Å, b = 5.74 Å, which are in accordance with those of previous works [46,47]. The calculated lattice constants for Si_GaI_ and Si_GaII_ structures are a = 2.98 Å, b = 5.77 Å and a = 2.99 Å, b = 5.76 Å, respectively. Only a slight structural difference was observed due to the small alteration in local structures and the similar ionic radii between the host Ga and the Si dopant. The formation energies for Si_GaI_ and Si_GaII_ structures are −5.41 and −6.27 eV, respectively, under O-rich conditions, which demonstrates that the Si dopant preferentially incorporates on the tetrahedrally coordinated GaII site. This is consistent with the result of Ref. [31]. Moreover, Si_GaI_ and Si_GaII_ structures possess the formation energies of −0.98 and −1.84 eV under a Ga-rich environment, respectively, illustrating that the foreign Si atom also prefers to occupy the GaII site herein. The higher formation energies also suggest the difficulty of introducing Si dopant in a Ga-rich atmosphere. In terms of the lower formation energy of the Si_GaII_ configuration compared with that of Si_GaI_, we merely studied the Si_GaII_ case under O-rich conditions afterwards. The phonon dispersion spectrum and AIMD simulation of the Si_GaII_ structure are employed in Figure S1a,b (supporting materials), indicating the dynamical stability and thermodynamical stability of the Si_GaII_ system, respectively.

### 3.2. Strain-Engineered Band Structures

**Figure 2:**
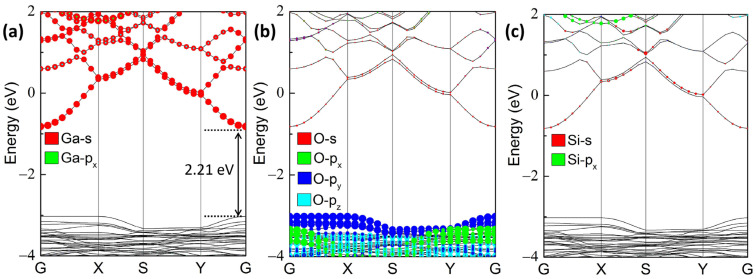
Orbital-projected band structures of (**a**) Ga, (**b**) O, and (**c**) Si orbitals for Si_GaII_ without strain. The strengths of the contributions are illustrated by the colored belt.

Figure 1b shows the band structure of perfect 2D β-Ga_2_O_3_, where the CBM is situated at the G point, whereas the VBM is positioned between the G and X points, demonstrating an indirect band gap semiconductor character. The calculated band gap of 2D β-Ga_2_O_3_ is 2.30 eV, which agrees well with previous works [45,47], but it is lower than the value of 3D β-Ga_2_O_3_ [48,49]. As shown in Figure 2, after substituting the tetrahedrally coordinated GaII with one Si atom, the band gap was slightly reduced to 2.21 eV. Moreover, the shift of VBM from the non-G point (between the G and X points) to the G point resulted in the direct band gap nature. These observations are consistent with the results in ref. [31]. Additionally, the orbital-projected band structures in Figure 2a–c illustrate that the majority of the VBM of Si_GaI_ is composed of O-2p orbitals, whilst the CBM is mainly contributed by Ga-4s and small quantities of O-2s and Si-3s orbitals.

**Figure 3:**
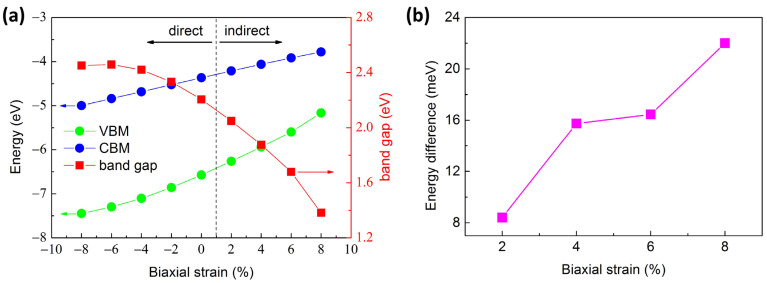
(**a**) Evolutions of band edges relative to the vacuum level and band gaps of Si_GaII_ with respect to biaxial strains. (**b**) The energy differences between the indirect and direct band gap versus biaxial tensile strains in Si_GaII_ structure.

The variations in band edges relative to the vacuum level and the band gaps of Si_GaII_ versus biaxial strain are shown in Figure 3a. The band gaps of Si_GaII_ increased slightly at first followed by a rapid decrease when the strain was applied from −8% compressive to +8% tensile cases. We note that this strain interval is of research focus in 2D β-Ga_2_O_3_ systems theoretically, which can also be induced by the lattice mismatch, ideally using a different substrate. The orbital-projected band structures of Si_GaII_ in Figure 2 can be used to explore the evolution behaviors of band gaps caused by biaxial strain. The σ* anti-bonding states that predominated in the CBM of Si_GaII_ are formed by the exceptional Ga-s orbitals, tiny O-s, and Si-s orbitals; the π bonding states near VBM of Si_GaII_ are mainly occupied by O-p_y_ orbitals. In general, lengthening the π bonding and σ* anti-bonding (from compressive to tensile strains) gives rise to the energy increases in CBM and VBM, as shown in Figure 3a [50]. The changes in energy levels can be attributed to the introduction of the Si dopant, whose orbital energy levels are hybridized with the host Ga-S orbitals, resulting in band gap changes. Consequently, the bigger the energy elevation of the VBM relative to the CBM, the smaller the band gap.

When applying biaxial tensile strain, the VBM moved from the G point to the non-G point, which resulted in a direct-to-indirect band gap transition. According to our findings, the location of VBM is therefore more susceptible to biaxial tensile strain than to biaxial compressive strain. Moreover, the compressive strain is beneficial for maintaining a direct band gap in the Si_GaII_ structure. When biaxial strain was applied, the band gaps of Si_GaII_ were tunable from 2.46 eV (−8% compressive strain) to 1.38 eV (+8% tensile strain). The energy differences between the direct and indirect band gap with respect to the biaxial tensile strains are shown in Figure 3b. One can notice that the energy difference was increased from 8 to 22 meV as the tensile strains increased.

### 3.3. Strain-Engineered Transport Properties

**Figure 4:**
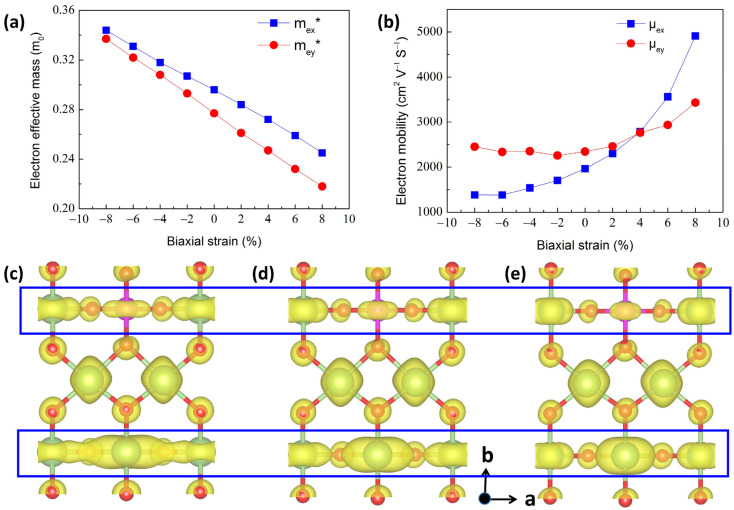
(**a**) Electron effective masses of Si_GaII_ versus biaxial strains. (**b**) Electron mobility of Si_GaII_ relative to biaxial strains. Partial charge distributions at the CBM for Si_GaII_ in three different strain cases: (**c**) 0%, (**d**) +4%, and (**e**) +8%. The isosurface level was measured at 0.001 e/Å^3^. The blue square box highlights the changes of the wave-function of the CBM along a direction.

The fluctuations of electronic structures with different strains were further employed to determine the carrier mobility (μ) and effective masses (m*). The electron effective masses (m_e_*) along the G–X and G–Y directions are labeled m_ex_* and m_ey_*, respectively. The calculated m_ex_* and m_ey_* of perfect 2D β-Ga_2_O_3_ are 0.29 m_0_ and 0.27 m_0_, respectively, which is in good agreement with the results of 0.31 m_0_ and 0.29 m_0_ in Ref. [51], as well as 0.36 m_0_ and 0.36 m_0_ (isotropy) in Ref. [52]. For unstrained Si_GaII_, the calculated m_ex_* and m_ey_* are 0.30 m_0_ and 0.28 m_0_, respectively, as shown in Figure 4a. The detailed variations in m_ex_* and m_ey_* under biaxial strains are exhibited in Figure 4a. It can be shown that the m_ex_* and m_ey_* significantly decreased from biaxial compressive to tensile strains. The small m_e_* generally implied a higher electron mobility (μ_e_). The m_ey_* decreased faster than m_ex_* with the transition of biaxial strains from compressive to tensile. Moreover, the anisotropy of the m_e_* was also enhanced when applying the biaxial tensile strains.

Different strains ranging from −4% to 4% were employed to calculate the elastic constants (C_2D_). Figure S2a in the supporting materials shows that the C_2Dx_ and C_2Dy_ of Si_GaII_ are 204.57 and 181.61 N m^−1^, respectively, smaller than the theoretical values of 324.18 and 329.08 N m^−1^ [53], and 333.2 and 330 N m^−1^ [54], as well as experimental values of 343.8 and 347.4 N m^−1^ [55] in 3D β-Ga_2_O_3_, respectively. This demonstrates that 2D Si_GaII_ is softer and more susceptible to deformation than bulk β-Ga_2_O_3_ material and may have greater potential for applications in flexible electronics. Figure S2b in the supporting materials denotes the calculated deformation potential E_1_ of the 2D Si_GaII_ structure. The E_1x_ and E_1y_ of the unstrained 2D Si_GaII_ structure are 4.11 and 3.79 eV, respectively. Accompanying the transition of biaxial stains from compressive to tensile, the E_1x_ of Si_GaI_ increased initially and subsequently decreased, while the E_1y_ increased monotonously. Figure S3 (Supporting Materials) shows the calculated details of E_1_.

The electron mobility was calculated based on the DP theory, and the changes in electron mobility are mainly dependent on the variations in m_e_*, E_1_, and C_2D_. Figure 4b illustrates the μ_e_ of 2D Si_GaII_ versus the biaxial strains at 300 K, where the μ_ex_ increased significantly and the μ_ey_ decreased slightly first and rose from compressive to tensile strains. Therefore, when the biaxial tensile strain was higher than 4%, it had a higher variation in μ_ex_ than μ_ey_, which can be associated with the smaller E_1x_ of CBM, as depicted in Figure S2b. When the biaxial tensile strain was lower than 4% or under compressive strain, the *y* direction possessed a higher μ_e_. The highest μ_ex_ and μ_ey_ were 4911.183 and 3434.44 cm^2^ V^−1^ s^−1^ as the tensile strain reached 8%, which was far greater than those reported in other doped 2D β-Ga_2_O_3_, i.e., H dopant with, respectively, 2684.93 cm^2^ V^−1^ s^−1^ and 156.25 cm^2^ V^−1^ s^−1^ along the *x* and *y* directions, F of ~300 cm^2^ V^−1^ s^−1^, Cl and H co-dopant of ~100 cm^2^ V^−1^ s^−1^ [56], as well as the H and F co-dopant in our previous work with 4863.05 cm^2^ V^−1^ s^−1^ under +6% uniaxial tensile strain along the *x* direction and 2175.37 cm^2^ V^−1^ s^−1^ under +4% uniaxial tensile strain along the *y* direction [43]. Nevertheless, the μ_e_ tendency was not saturated, which demonstrated that a higher μ_e_ was expected under a larger biaxial strain above 8%. It is noteworthy that, despite the high compressive strain (up to −8%), the μ_e_ remained greater than 1300 cm^2^ V^−1^ s^−1^, which surpassed the μ_e_ in other monolayer materials, such as α-In_2_Se_3_ (~1000 cm^2^ V^−1^ s^−1^) [57], MoS_2_ (~200 cm^2^ V^−1^ s^−1^) [58], and GaN (~300 cm^2^ V^−1^ s^−1^) [59]. Thus, in terms of its exceptional μ_e_, the 2D Si-doped β-Ga_2_O_3_ is highly promising for applications in nanoscale optoelectronic devices.

The spatial charge distributions of the unstrained Si_GaII_ structure, as illustrated by the yellow regions, was employed to explain the changed mechanism of electron mobility, as shown in Figure 4c. The wave-function of the CBM is largely embedded in the surface along the a direction, as marked by the blue square box. This means that the production of high μ_e_ is inhibited by the strong quantum confinement effect on the surface. When the biaxial tensile strains were boosted from +4% to +8%, as depicted in Figure 4d and Figure 4e, respectively, the quantum confinement effect became weaker, giving rise to an enhancement of μ_e_.

We note that biaxial strains can be commonly induced by the lattice mismatch between the substrate and the film according to the following equation: *mismatch* = (*a_film_* − *a_substrate_*)/*a_substrate_*. Taking different substrates such as sapphire, SiC, Si(111), GaN, and AlN with the same hexagonal symmetry into account, we can calculate that the ideal strains induced by sapphire, SiC, Si(111), GaN, and AlN along the direction of lattice a are 8.85%, −2.95%, −4.63%, −6.24%, and −3.89%, respectively, and along rhe direction of lattice b, they are 4.84%, −6.52%, −8.13%, −9.65%, and −7.43%, respectively. We further note that for β-Ga_2_O_3_ on sapphire substrate, the 30° domain structure was considered from the crystallographic point of view [60]. Our calculated results predict that the highest electron mobility is expected in Si-doped β-Ga_2_O_3_ deposited on the sapphire.

**Figure 5:**
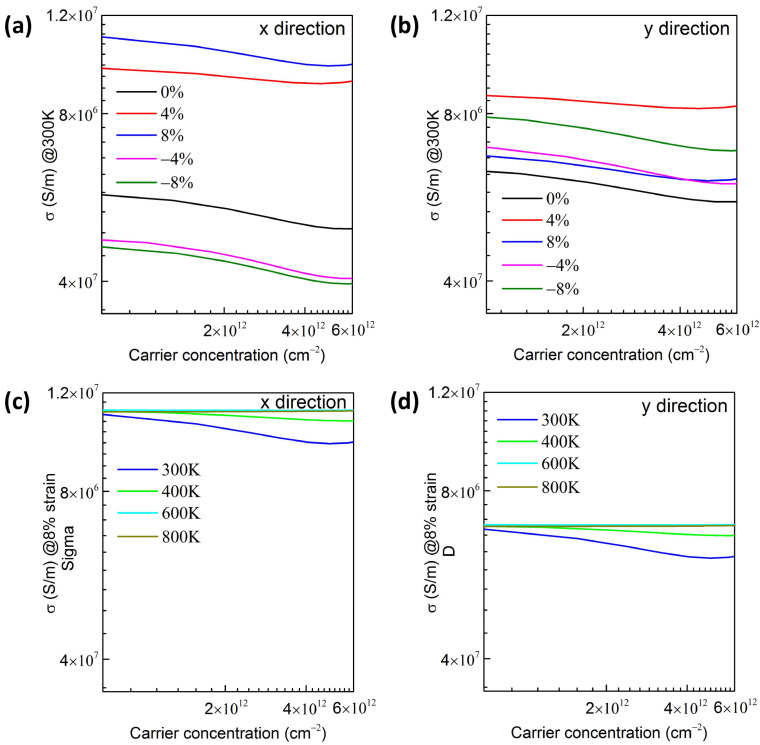
The σ for n-type Si_GaII_ configuration at 300 K along (**a**) *x* direction and (**b**) *y* direction relative to carrier concentration. The σ of Si_GaII_ configuration with 8% strain along (**c**) *x* direction and (**d**) *y* direction with different temperatures.

Figure S4 (supporting materials) shows the relaxation time τ of the Si_GaII_ structure, which possesses the same tendency as μ_e_. The electrical conductivity (σ) of the Si_GaII_ structure is acquired base on the τ and BoltzTraP2 code [42,61]. Figure 5a and Figure 5b show the σ with respect to n-type carrier concentrations at the temperature of 300 K with varying strains along the *x* and *y* directions, respectively. Along the *x* direction, when the doping concentrations varied from 7 × 10^11^ to 6 × 10^12^ cm^−2^ at 300 K, the electron mobilities of unstrained and +4%, +8%, −4%, and −8% strained Si_GaII_ ranged from 5.69 × 10^6^ to 4.97 × 10^6^ cm^2^ V^−1^ s^−1^, 9.76 × 10^6^ to 9.29 × 10^6^ cm^2^ V^−1^ s^−1^, 1.10 × 10^7^ to 9.83 × 10^6^ cm^2^ V^−1^ s^−1^, 4.78 × 10^6^ to 4.05 × 10^6^ cm^2^ V^−1^ s^−1^, and 4.66 × 10^6^ to 4.01 × 10^6^ cm^2^ V^−1^ s^−1^, respectively. Therefore, the σ was increased when applying the tensile strains, and declined when applying the compressive strains, which is in accordance with the changing trends of μ_ex_ in Figure 4b. However, different from the trends of μ_ey_ in Figure 4b, the σ was increased when applying the tensile or compressive strain along the *y* direction. Moreover, the 2D Si_GaII_ with +4% tensile strain possesses the highest σ along the *y* direction. Motivated by the outstanding μ and σ obtained in the +8% strained Si_GaII_, the σ values with respect to the different temperatures were considered as well. The fluctuations in σ at different carrier concentrations for +8% strained Si_GaII_ along the *x* direction are depicted in Figure 5c, which first boosted quickly and then remained essentially steady from 300 K to 800 K. Figure 5d shows the σ along the *y* direction, which exhibits the same trends as those in Figure 5c.

### 3.4. Conclusions

Using first-principles methods in conjunction with deformation potential (DP) theory and BoltzTraP2 code, we systematically investigated the Si-doped 2D β-Ga_2_O_3_ structure mediated by biaxial strain. The structural stabilities of Si-doped 2D β-Ga_2_O_3_ were determined by formation energies, phonon spectrum, as well as AIMD simulations. The band gap values of Si-doped 2D β-Ga_2_O_3_ initially increased slightly, followed by a rapid decrease from 2.46 eV to 1.38 eV accompanied by strain modulations from −8% compressive to +8% tensile, which can be ascribed to the bigger energy elevation of the σ* anti-bonding in the CBM than that of π bonding in the VBM. Additionally, band structure calculations resolved a direct-to-indirect transition under the tensile strains. The μ_ex_ increased significantly and the μ_ey_ decreased slightly first and rose from compressive to tensile strain. When the biaxial tensile strain was higher than 4%, it had a higher variation in μ_ex_ than in μ_ey_, which can be associated with the smaller E_1x_ of CBM. When the biaxial tensile strain was lower than 4% or under compressive strain, the *y* direction possessed a higher μ_e_. The highest μ_ex_ and μ_ey_ were 4911.183 and 3434.44 cm^2^ V^−1^ s^−1^ as the biaxial tensile strain approached 8%, which was far greater than those reported in other doped 2D β-Ga_2_O_3_. The electrical conductivity was elevated with the increase in tensile strain and the enhancement of temperature from 300 K to 800 K. Our work demonstrates that the electron mobilities and band structures of Si-doped 2D β-Ga_2_O_3_ are tunable by biaxial strain, highlighting its great potential in nanoscale electronics.

## Data Availability

The raw data supporting the conclusions of this article will be made available by the authors on request.

## Supplementary Materials

The following supporting information can be downloaded at: https://www.mdpi.com/article/10.3390/ma17164008/s1, Figure S1: (a) Phonon dispersion and (b) AIMD simulation for the 6 × 2 × 1 supercell of the Si_GaII_ under O-rich condition at the temperature of 300 K. The inserted plot in Figure S1b indicates the optimized lattice structure of SiGaII; Figure S2: (a) Total energies of 2D Si_GaII_ with respect to strain, and the in-plane elastic constant C_2D_ are also added. (b) Deformation potentials of 2D Si_GaII_ versus uniaxial strains; Figure S3: Energy shift of CBM of 2D Si_GaII_ under uniaxial strains along *x* (a,b) and *y* (c,d) directions, respectively. The slope indicates the deformation potential constant E_l_ of CBM; Figure S4: The relaxation time τ of 2D SiGaII with respect to different uniaxial strains.
