# Supplementary material for: High Electron Mobility in Si-Doped Two-Dimensional β-Ga2O3 Tuned Using Biaxial Strain
Source: Materials (Basel). 2024 Aug 12;17(16):4008. doi: 10.3390/ma17164008 (PMC11356731; doi:10.3390/ma17164008)
Supplement: Supplementary file 1 [file materials-17-04008-s001.zip › materials-3105919-supplementary.pdf]

High Electron Mobility in Si-Doped Two-Dimensional β-Ga_2O_3 Tuned Using Biaxial Strain

Hui Zeng [1,2,*], Chao Ma [2] and Meng Wu [3,*]

[1] College of Science, Hunan University of Science and Engineering, Yongzhou 425199, China

[2] College of Materials Science and Engineering, Hunan University, Changsha 410082, China

[3] Fujian Provincial Key Laboratory of Semiconductors and Applications, Collaborative Innovation Center for Optoelectronic Semiconductors and Efficient Devices, Department of Physics, Xiamen University, Xiamen 361005, China

***** Correspondence: 19820170155498@stu.xmu.edu.cn (H.Z.); meng.wu@xmu.edu.cn (M.W.)

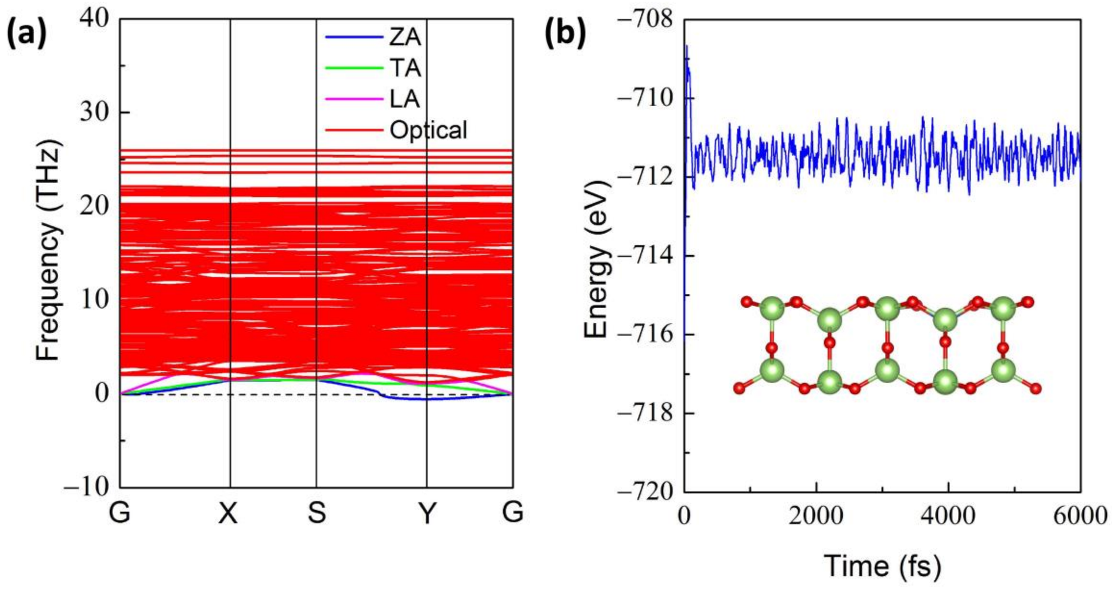

Figure S1 (a) Phonon dispersion and (b) AIMD simulation for the 6×2×1 supercell of the Si_{GaII} under O-rich condition at the temperature of 300 K. The inserted plot in Figure S1(b) indicates the optimized lattice structure of Si_{GaII}.

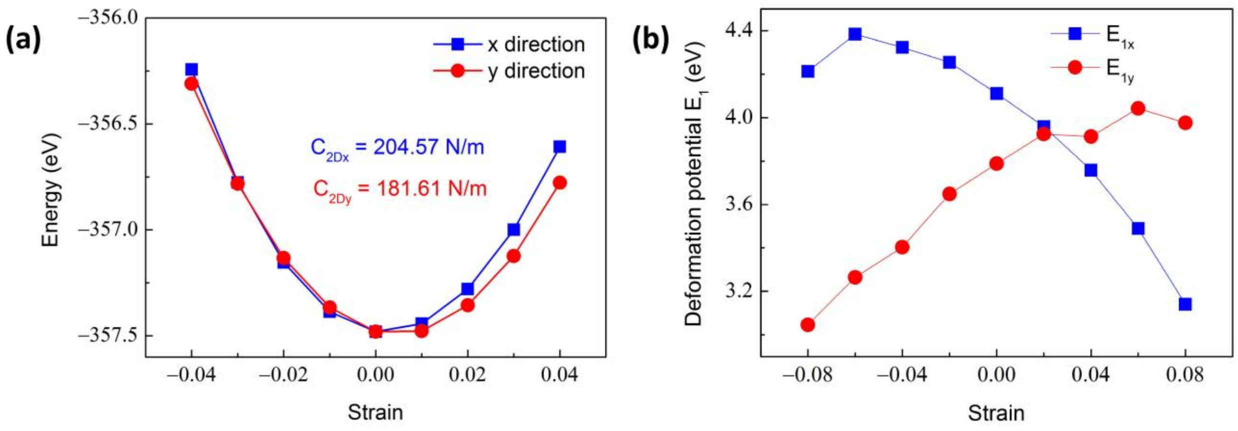

Figure S2 (a) Total energies of 2D Si_{GaII} with respect to strain, and the in-plane elastic constant C_{2D} are also added. (b) Deformation potentials of 2D Si_{GaII} versus uniaxial strains.

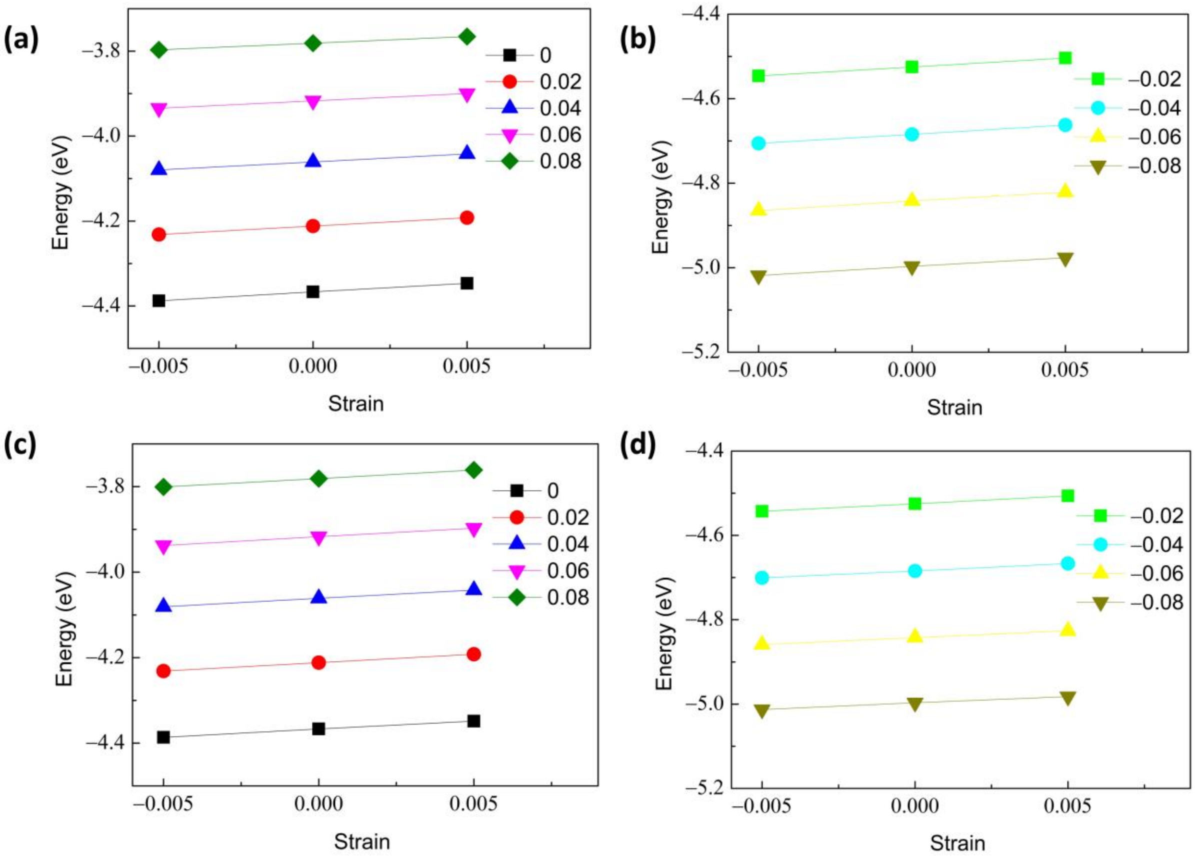

Figure S3 Energy shift of CBM of 2D Si_{GaII} under uniaxial strains along *x* (a-b) and *y* (c-d) directions, respectively. The slope indicates the deformation potential constant E_l of CBM.

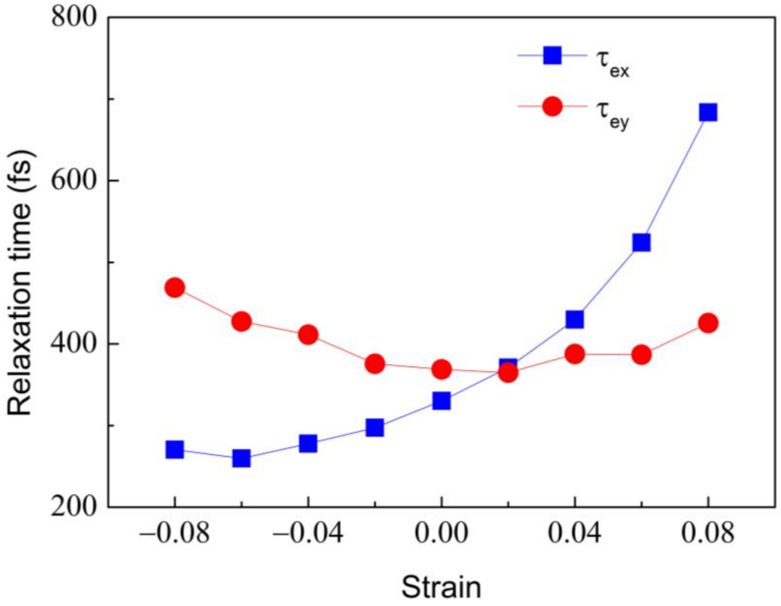

Figure S4 The relaxation time τ of 2D Si_{GaII} with respect to different uniaxial strains.
